# Piano Education of Children Using Musical Instrument Recognition and Deep Learning Technologies Under the Educational Psychology

**DOI:** 10.3389/fpsyg.2021.705116

**Published:** 2021-09-16

**Authors:** Huizi Li

**Affiliations:** School of Music and Recording Arts, Communication University of China, Beijing, China

**Keywords:** piano education, educational psychology, children's quality education, deep learning, musical instrument recognition

## Abstract

The objective of the study was to enhance quality education in the traditional pre-school piano education. Deep Learning (DL) technology is applied to piano education of children to improve their interest in learning music. Firstly, the problems of the traditional piano education of children were analyzed with the teaching patterns discussed under educational psychology, and a targeted music education plan was established. Secondly, musical instrument recognition technology was introduced, and the musical instrument recognition model was implemented based on DL. Thirdly, the proposed model was applied to the piano education of children to guide the music learning of students and improve their interest in piano learning. The feature recognition and acquisition of the proposed model were improved. Finally, the different teaching patterns were comparatively analyzed through the Questionnaire Survey (QS). The experimental results showed that the instrument recognition accuracy of Hybrid Neural Network (HNN) is 97.2%, and with the increase of iterations, the recognition error rate of the model decreases and stabilizes. Therefore, the proposed HNN based on DL for musical instrument recognition can accurately identify musical features. The QS results showed that the introduction of musical instrument recognition technology in the piano education of children can improve their interest in piano learning. Therefore, the establishment of the piano education patterns based on the piano education model can improve the effectiveness of teaching piano to students. This research provides a reference for the intelligentization of children's piano education.

## Introduction

With social development, more attention is being paid to the quality education of children. Music education can cultivate the sentiments of children, promote the healthy development of their body and mind, and strengthen perseverance (Fan, 2019). The purpose of pre-school education for children is to improve the cultural accomplishment of children and their overall quality through emotional music. Therefore, piano education can be used as a teaching content of quality education for children. However, children are starting to learn the piano at a young age and therefore, may easily lose interest in learning during the boring piano practice (Gerelus et al., 2020). Therefore, teachers should design scientific piano learning strategies that can manifest the charm of piano performance for children according to their age, the relevant contents of pedagogy, and educational psychology (Qian et al., 2018). Through the introduction of deep learning (DL) technology into piano education, targeted teaching programs can be designed for different students, and with the combination of psychological problems of children, each child can always be closely attended to Chen et al. (2021). Here, to enhance the teaching ability of the piano education of children, musical instrument recognition technology is established with the DL algorithm and applied to the piano education of children to promote the intellectualization of piano education.

Domestic and international scholars have rigorously researched piano education. Shuo and Xiao (2019) studied piano education through the intelligent network, established a piano note recognition algorithm, and implemented it in piano education. Some piano audio files were transcribed, tested, and annotated to construct a linear annotation recognition model (Shuo and Xiao, 2019). Liu (2021) explored the interest of parents in the piano education of their children through personal experiences and the status of piano education and compared and analyzed some Chinese and foreign piano education methods (Liu, 2021). Mei and Yang (2021) applied Augmented Reality (AR) technology in piano education, conducted case analysis through experiments, and collected experience for piano education based on AR technology. The survey results showed that the piano education method based on AR technology had specific practical value (Mei and Yang, 2021). Blasco-Magraner et al. (2021) studied the importance of emotional ability in music teaching and examined the positive impact of music on the emotions of children aged 3–12. The experimental results showed that music had a positive impact on the emotional quotient (EQ) development of the children, academic performance, and social skills enhancement (Blasco-Magraner et al., 2021).

Here, the instrument recognition technology based on DL was applied to the piano education of children to improve their piano learning interest, thereby improving the teaching effect of piano education and strengthening quality education for children. Firstly, the current situation of the piano education of children was analyzed. Combined with the relevant content of educational psychology, curriculum innovation was carried out, and the application of instrument recognition technology in the piano education of children was proposed. Afterward, the music recognition technology was designed based on DL, and then a QS was issued to explore the influence of teaching patterns on the piano education of children. Innovatively, DL was applied to the piano education of children to improve their interest in learning piano, identify the musical works of the children through the designed DL learning system, guide and adjust the timeliness of the piano performance of children, and stimulate the initiative of students for learning the piano.

## Application of DL In Piano Education of Children

### Piano Education of Children Under Educational Psychology

In traditional piano education, a teacher may teach one to tens of students simultaneously. Therefore, not all the performances can be observed and guided timely. Thus, student number is strictly controlled to ensure an active classroom atmosphere and to attend to each student (Barsamyan, 2019; Wu and Song, 2019). Traditionally, teachers focus on course contents, and students assimilate and digest what is taught. Students are passively involved both in theories and playing techniques and therefore, can hardly perceive the essence of the piano performance. Consequently, they may lack the initiative to learn to play (Rogoza et al., 2018; Burrows and Brown, 2019). Therefore, piano education children should be innovated and deepened, and teachers should awaken the interest of the children in learning and performance through a novel and humorous course design (Chen, 2019; Wu et al., 2019; Li and Tian, 2020).

Meanwhile, it has been argued that children benefit from pleasant classroom environments, and improvement of performance skills should not solely rely on repeated exercises (Mierowsky et al., 2020; Sabljar et al., 2020). Self-control and willpower of children are relatively weak, so the role of parents in their piano practice cannot be overemphasized. This makes parental companionship important throughout the course. Thus, parents and teachers should collaborate harmoniously, and teachers should innovate customized piano courses based on the educational psychology of children, thereby catering to the healthy development of their mentality (Hamond et al., 2019).

People perceive knowledge through cognitive activities, and in the piano education of children, psychological activities should be analyzed (Guhn et al., 2020). Educational psychology can provide a basis for the educational mental health of children to optimize teaching methods.

According to the growth status and ages of children, they can be divided into different groups: infancy, early childhood, preschool, school age, and adolescence. Here, children younger than seven are classified into pre-schoolers. Some of these pre-schoolers, aged 4–6 years old, are recruited for experimental education.

It is believed that the intelligence of children develops the best in early childhood. In line with this, pre-school education can help children cultivate and improve the overall quality of their intellect. Music can provide pre-schoolers, aged 4–6, with skills to display their imagination and performance. Children are often keen to create and play in the music world, which can bring them the freshness and fun of music performance (Woody, 2020). Therefore, teachers should refresh teaching techniques to help students learn the theoretical knowledge and skills necessary in piano performance.

### Training Musical Sense of Children Through Musical Instrument Recognition Technology

Piano education of children focuses on personality development to cultivate creativity in students. It has been argued that students can benefit from targeted piano courses based on their personalities and be cultivated comprehensively. Piano music can shake hearts, and musical sense is the ability to perceive this feeling. It has been argued that children benefit from teaching strategies that can cultivate their musical sense from solfeggio and ear training which pays more attention to rhythm and tone. In the beginning, tone and spectrum awareness of children should be strengthened through constant singing and playing. Hearing should be trained through attentive listening on fixed melodies so that children can pick out any wrong tone in piano music and adjust themselves accordingly. Thus, a sense of accomplishment and the confidence of children will be exerted during piano practice (Guo and Cosaitis, 2020; Kurtuldu, 2021).

Instrument recognition technology can classify, annotate, and identify music emotions. It can also acquire instrument names, extract melodies, and annotate musical texts for music transcription and visualization. Additionally, it can extract the tones of the instrument to construct a new digital music evaluation model. The basis of musical instrument recognition is to distinguish instrumental tones. Computer Technology (CT) can distinguish instrumental tones more accurately. The effectiveness of the evaluation model depends on the tone analysis algorithm. Thus, the accurate extraction of instrumental tone is essential to CT instrument recognition (Comeau et al., 2019).

During instrumental music recognition, the timbre features should be represented. Most commonly, the Mel cepstrum feature, orthogonal matching, timbre descriptor, and probability feature representation methods are used. Humans do not rely on the cepstrum to recognize musical instruments but process the sound signal into the auditory spectrum, reflects the harmonic information through the auditory spectrum, and process the time and frequency of the auditory spectrum by multi-scale video modulation, thereby obtaining the time-frequency information of instrumental music. Mel spectrum is proposed according to the auditory characteristics of human ears and has a non-linear relationship with frequency. Based on this relationship, the Mel Frequency Cepstrum Coefficient (MFCC), or the frequency spectrum characteristic, is calculated, which is mainly used for feature extraction of speech data and dimension reduction operations. The above instrumental music recognition methods belong to the shallow structure, and the difference lies in timbre feature extraction and classifier optimization. However, when dealing with complex problems, such models cannot explore the characteristics of music, such as percussion and orchestral instruments.

### Musical Instrument Recognition Based on DL

**Figure 1:**
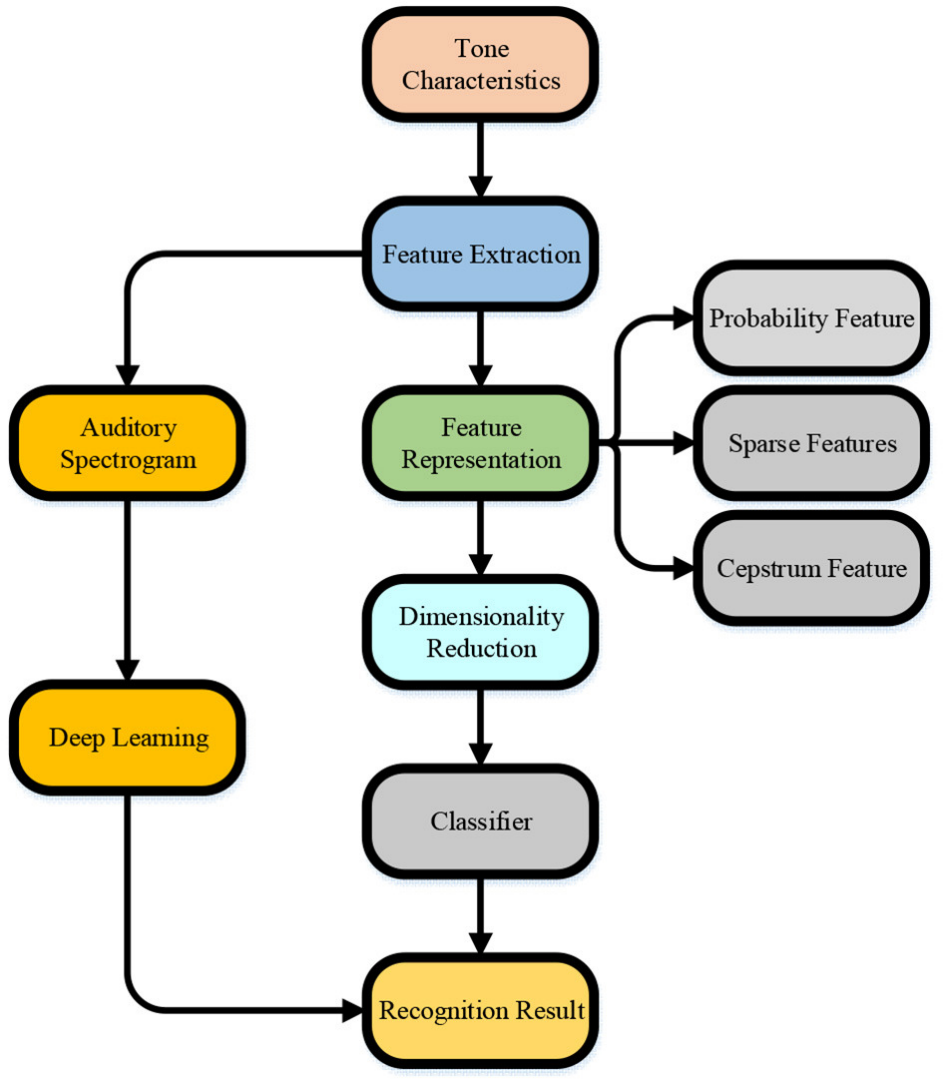
Technological process of musical instrument recognition.

The DL can extract and abstract information more accurately and learn musical instrument features at different levels. The input of the neural network affects the feature extraction efficiency and instrumental recognition accuracy. Hence, the auditory spectrogram is chosen for the input of the neural network because the auditory spectrum can better reflect the sound reception range of humans than the Spectrogram and can extract advanced sound features in the neural network (Yu et al., 2020). Figure 1 shows the technical process of musical instrument recognition.

**Figure 2:**
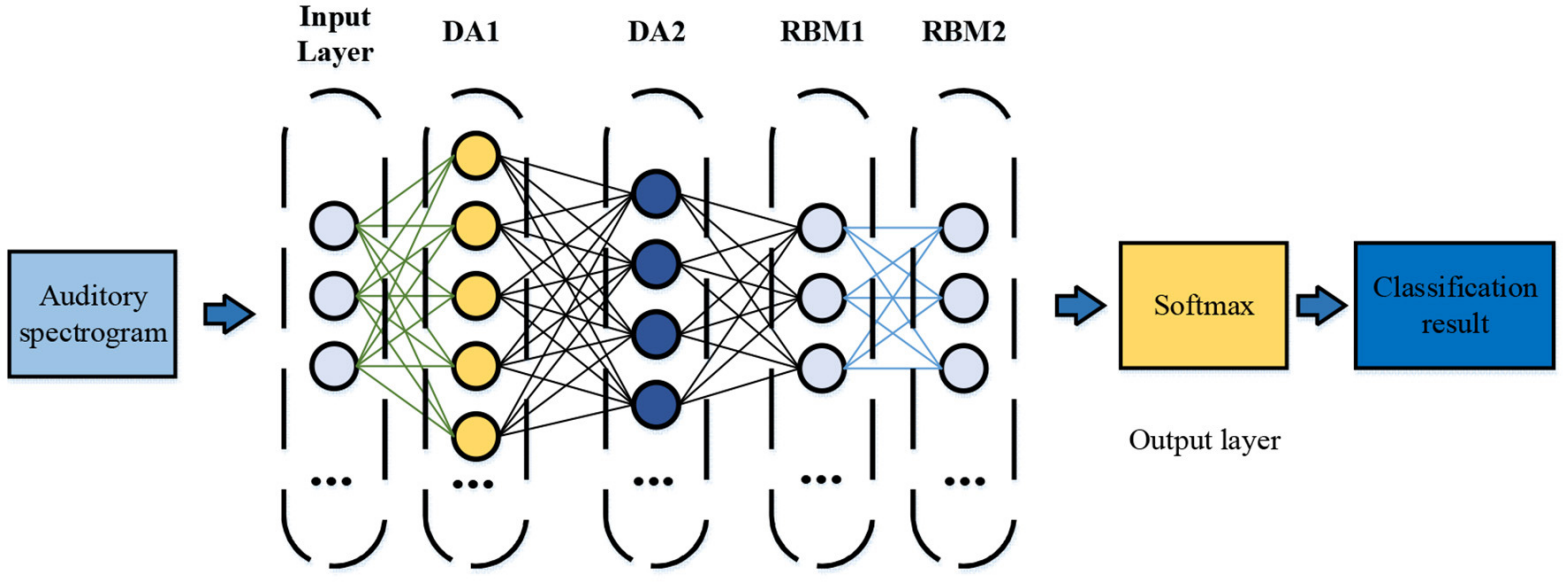
Hybrid Neural Network (HNN) structure of musical instrument recognition.

Here, the Denoise Autoencoder (DA) and the Restricted Boltzmann Machines (RBM) are concatenated to estimate the distribution of the input signal of the neural network and to enhance feature expression. Then, the Softmax layer is used as the classifier (Li et al., 2019). The DA can expand the projection of the original data and pre-train the RBM through the contrast three-degree method. Layer-by-layer pre-training ensures good initial weights for the Neural Network, and the neural network is fine-tuned through the Backpropagation (BP) algorithm, thus effectively identifying the instruments (Szenicer et al., 2019). Figure 2 shows the structure of the proposed Hybrid Neural Network (HNN) for musical instrument recognition based on DL and auditory spectrogram. The structure includes two DA layers, two RNM layers, and one Softmax layer, and the auditory spectrogram is used as the input.

The DA randomly zeroize the input nodes based on specific probability distribution to denoise the weight matrix and ensure the stability of the activation value between the explicit layer and the hidden layer. The objective function *L*(*x*) can be expressed as in Equation (1).


(1) $$\begin{array}{r} L\left(x\right)=\underset{W}{\arg\min}||\sigma \left(W^{T}\sigma \left(W\tilde{x}\right)\right)-x|{|}_{2} \end{array}$$

In Equation (1), σ represents the sigmoid activation function, *W* denotes the connection weight matrix between the explicit layer and the hidden layer, $\tilde{x}$ stands for the explicit layer output after some elements are zeroized, and is the original data. The damaged data is $\tilde{x}$ output to the hidden layer, and the error is iterated through the original data x to denoise of the weight matrix, thereby reducing the error of the training data and the test data. The DA feature extraction from auditory spectrogram is enhanced through the configuration of hidden layer nodes number, facilitating abstraction of musical instrument features through RBM. Therefore, the original data is expanded in DA, abstracted in the second layer, and the output data in the dimension of the first layer is reduced and compressed (Xu et al., 2019).

**Figure 3:**
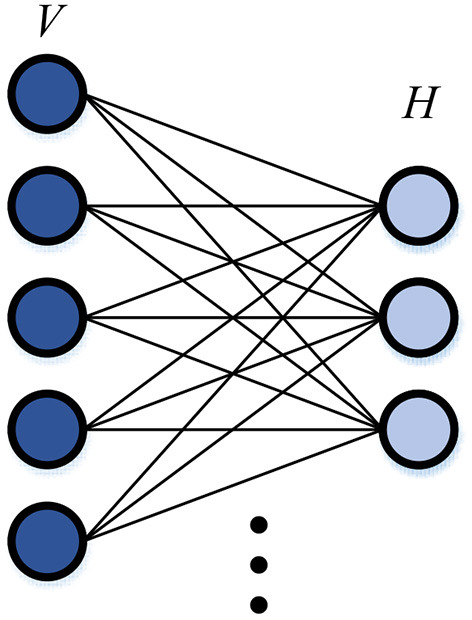
Structure of RBM.

The RBM is a generative stochastic neural network composed of visible units and hidden units, the model structure is shown in Figure 3. Both visible variable *v* and hidden variable are binary variables, that is, their states fall into {0, 1}. The Deep Belief Network (DBN) can be obtained through cascaded RBMs. The network is a bipartite gra*h*ph, and there is only a connection between the visible unit and the hidden unit. There is no connection between the visible units or the hidden units.

In the RBM, the joint probability distribution of the explicit layer and the hidden layer can be obtained through the parameters, such as the weight matrix and bias of the model, as expressed in Equation (2) and Equation (3), respectively.


(2) $$\begin{array}{r} p\left(v,h;\theta \right)=\frac{\exp\left(-E\left(v,h;\theta \right)\right)}{Z}\theta Z \end{array}$$

(3) $$\begin{array}{r} (Z=\underset{v}{\sum }\underset{h}{\sum }\exp\left(-E\left(v,h;\theta \right)\right) \end{array}$$

In Equation (2) and (3), *Z* represents the normalization factor, and θ denotes the model parameters. *E*(*v, h*; θ) stands for the energy function of the RBM network, which obeys the steady-state distribution. The value of the energy function is determined by the distribution of the input layer. The input of the first layer of DA follows the normal distribution, and the input value of the second layer of DA follows the Bernoulli distribution (Yu et al., 2019). The RBM network can distribute nodes in the explicit layer within the distribution space of the input sample, and the objective function can be calculated through the maximum likelihood function lnL(v), as expressed in Equation (4).


(4) $$\begin{array}{r} lnL\left(v\right)=\underset{W,a,b}{\arg\min}\underset{v\in S}{\sum }\ln\left(p\left(v\right)\right) \end{array}$$

**Figure 4:**
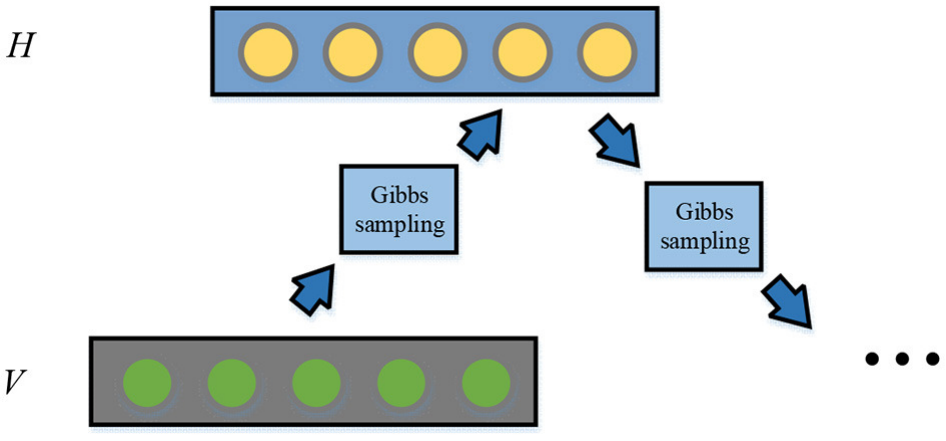
Flowchart of CD algorithm.

In Equation (4), S denotes the collection of input data. The Contrastive Divergence (CD) can initialize the training data, and then the spatial distribution of the samples is estimated. The gradient of the RBM parameters can be updated through the CD algorithm. The algorithm flowchart is shown in Figure 4.

**Figure 5:**
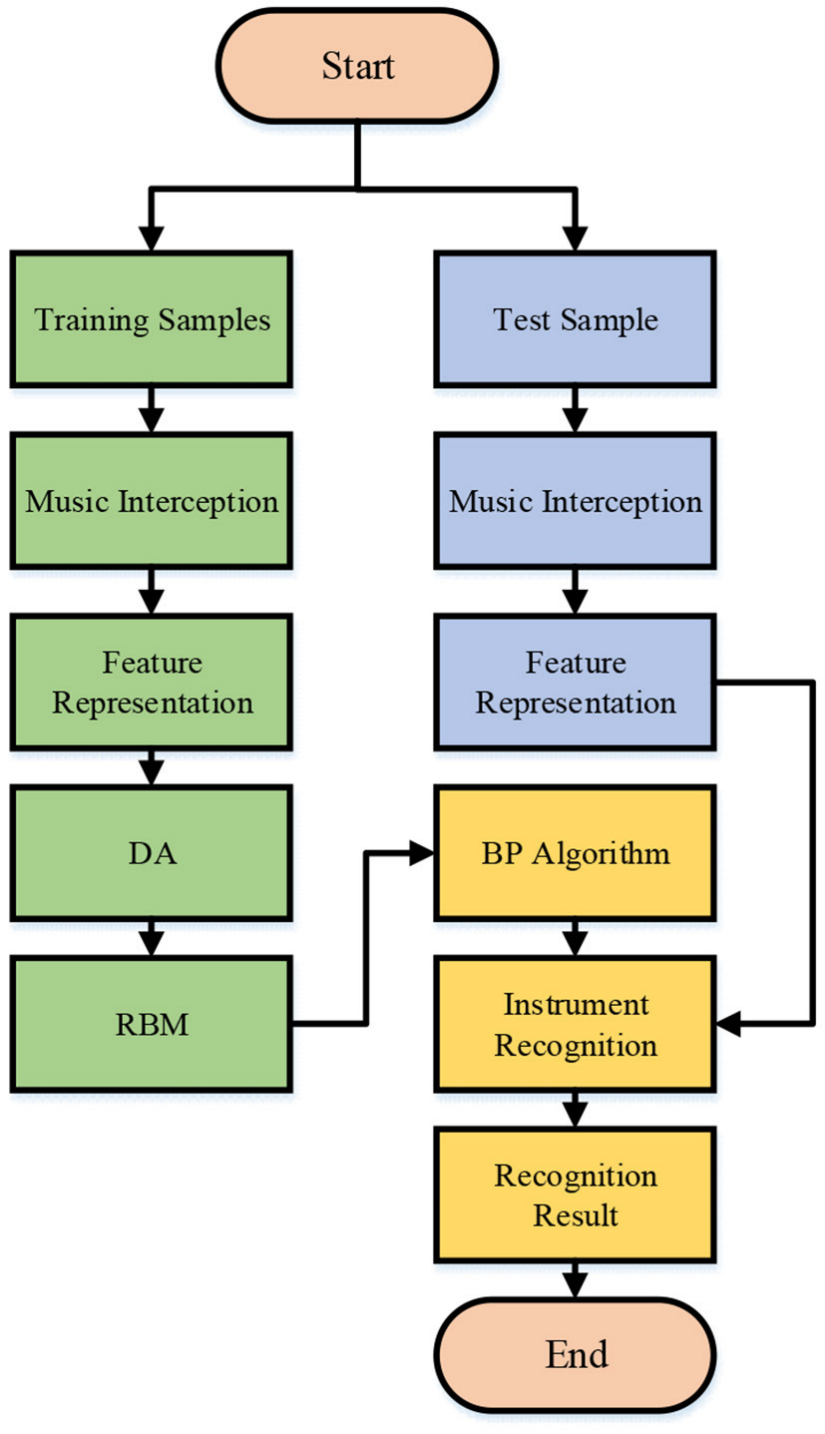
Musical instrument recognition process based on HNN.

The Learning Rate (LR) of HNN is calculated through Adaptive Moment Estimation (Adam) to avoid the slow convergence in HNN and loss function fluctuation caused by parameter updates. Adam algorithm combines the advantages of AdaGrad and RMSProp algorithms and calculates the parameter LR based on the one-order matrix and the two-order matrix (Ebigbo et al., 2019). The cross-entropy between the output of the Softmax layer and the data set is the objective function, and the network is accelerated through the Tensorflow framework. The identification process of musical instruments through the proposed HNN based on DL and auditory spectrogram is shown in Figure 5.

### QS Design and Reliability and Validity Test

Further, a QS is conducted from three levels of children, parents, and teachers to explore the application of intelligent piano based on DL in piano education of children, and the QS is designed by Likert type. The questions involved include three dimensions: (1) the QS of children includes their interest in intelligent piano and class status, (2) the QS of parents includes motivation, attitude, and advice on piano learning, and (3) the QS of teachers include their educational background, majors, teaching methods, and teaching strategies (Maguet et al., 2021). All three, together, constitute the whole QS. The experimental research is conducted upon the piano education institutions for children in X district of the city of Xi'an. The QS is mainly distributed through the online WeChat class group. Meanwhile, the QS star is used for the production, distribution, and recovery of the QS. Totally, 480 QSs are collected, meeting the survey standards. The issuance and recovery of the QS have been agreed upon by the parties, and the QS data are only used for related research. The detailed information of the questionnaire is shown in Table A1 in the appendix.

The research is exploratory, so relevant scales should be designed to analyze the problems. Specifically, SPSS 25.0 is used for statistical analysis of the collected QS data, and the scale is verified through the Exploratory Factor Analysis (EFA), while the validity of the scale is evaluated with Kaiser–Meyer–Olkin (KMO) test and Bartlett's sphericity test.

Generally, it is believed that a KMO value of above 0.9 in the validity evaluation indicates that the data are suitable for factor analysis. A KMO value between 0.8 and 0.9 suggests very suitable, a KMO value between 0.7 and 0.8 represents suitable, a KMO value between 0.6 and 0.7 means acceptable, a KMO value between 0.5 and 0.6 is very poor, and a KMO below 0.45 implies that the results should be abandoned. Here, the results of the KMO and Bartlett's sphericity tests are shown in Table 1. Obviously, the KMO value is 0.934 which means that validity is good and the data is very suitable for factor analysis. Chi-square value of Bartlett's sphericity test is 13321.772, *p* < 0.01, the data are very suitable for factor analysis. Further, the two common factors are rotated to explain the original variables more accurately with the extracted factors and increase the correlation between the common factors and the original variables (Küçük, 2020). Table 2 suggests that the data of the designed QS can reflect the actual problems and meet the needs of scientific research.

**Table 1:** KMO and Bartlett's sphericity tests.

KMO sample test quantity	0.934	
Bartlett's sphericity test	Approximate chi-square	13321.772
	df	12
	Sig.	0.000

**Table 2:** Rotational component matrix.

**Index**	**Component 1**	**Component 2**
1	0.782	0.670
2	0.808	0.672
3	0.771	0.534
4	0.870	0.780
5	0.879	0.872
6	0.892	0.673
7	0.769	0.587
8	0.939	0.975
9	0.638	0.876
10	0.980	0.575
11	0.909	0.568
12	0.569	0.867

### Experimental Data Set and Model Parameter Setting

Here, the experimental sample set is the audio library of the University of Iowa Electronic Music Studio, in which the instruments of various manufacturers and the monophonic parts of different players are recorded. The samples of instruments include a total of nine instruments, such as woodwind instruments, brass instruments, plucked string instruments, hammered string instruments, bowed string instruments, and percussion instruments. In the experiment, the bassoon, saxophone, tuba, flute, guitar, piano, violin, cello, and xylophone are selected as the experimental research options. The sample number of each instrument is 500, the training samples are 16-bit single-channel digital signals, and the sampling frequency is 44.1 kHz. In the sample library, 80% of the samples are randomly selected as the training set, and the remaining samples constitute the test set. All the samples are transformed into the auditory spectrum. The duration of the sample is 2 s, and the auditory spectrum is a 128 100 matrix. In the training process, the neural network with Back Propagation (BP) algorithm is fine-tuned with the training set, and the cycle traverses 50 times. The descent rate is adjusted with the Ada× m algorithm, and the step size of the gradient descent algorithm is set to 0.001. Experiment-related hyperparameter settings are shown in Table 3.

**Table 3:** Hyperparameters setting.

**Parameters**	**Value**	**Parameters**	**Value**
Learning rate	0.3	Network layers	6
Maximum training times	100	Weight change increment	1.2
Requirements for training accuracy	0.01	Weight change reduction	0.5
Minimum gradient requirements	1.00E-10	Changes in initial weights	0.07
Displays the training iteration process	25	Maximum value of weight change	50
Maximum training time	inf	Gradient descent algorithms	Adam algorithm
Momentum factor	0.9	Loss function	Quadratic mean square function
One-dimensional linear search method	srchcha	Activation function	Sigmoid function

## Test and QS Analysis

### HNN Test Analysis

**Figure 6:**
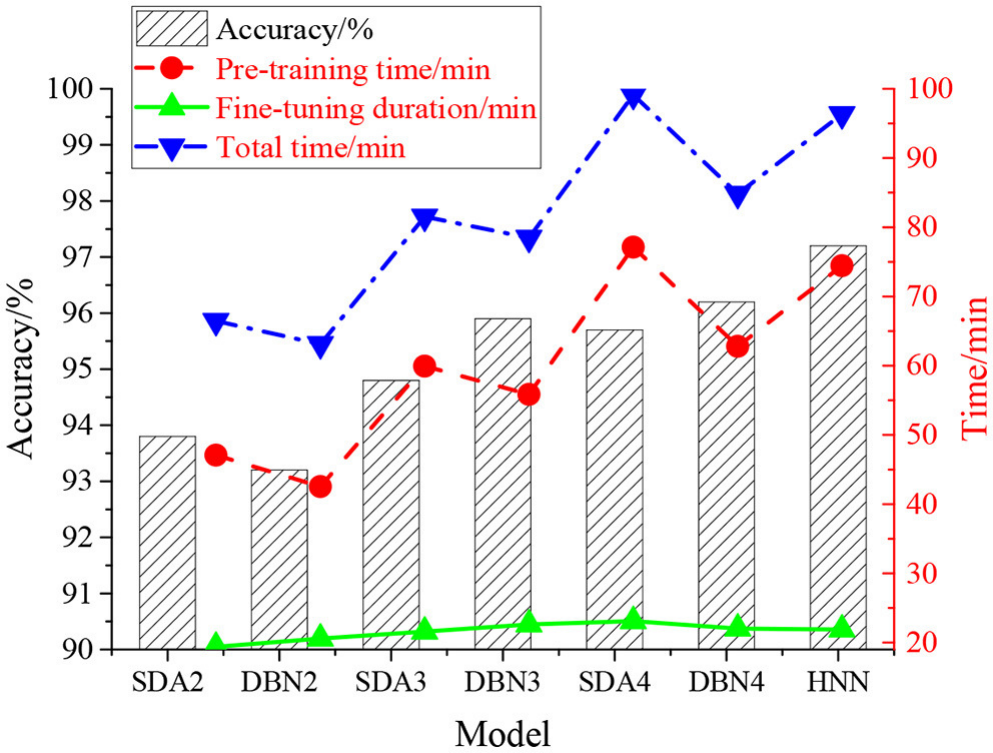
Model accuracy comparison.

The proposed model is compared with other neural network models with the same training parameters to verify the music recognition accuracy of the proposed HNN based on DL. The results are shown in Figure 6.

**Figure 7:**
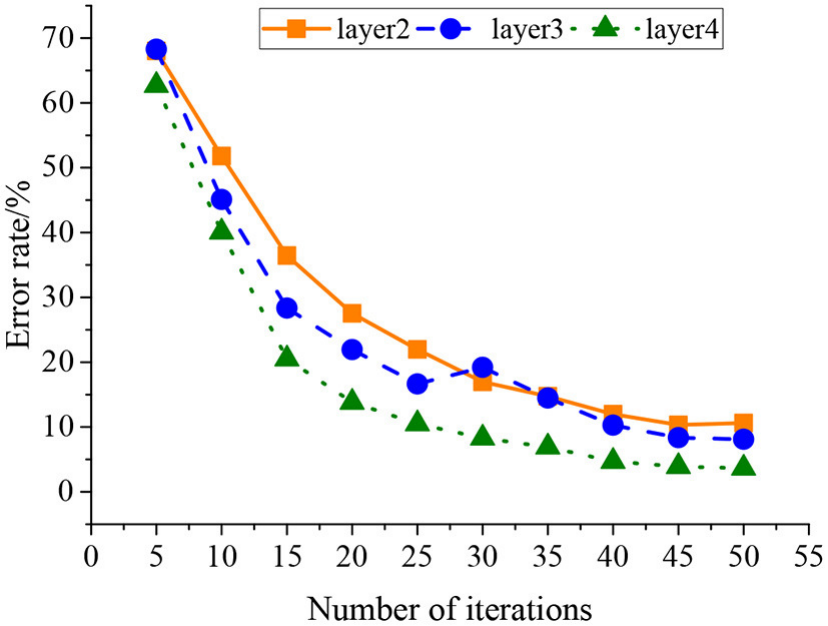
The training process of each layer of the HNN model.

The neural network model with different numbers of DA layers and RBM layers is chosen as comparison models, including SDA2 (Stacked Denoising Autoencoders) (neural network containing two DA layers, zero RBM layer, and one Softmax layer), DBN2 (neural network containing zero DA layer, two RBM layers, and one Softmax layer), SDA3 (neural network containing three DA layers, zero RBM layer, and one Softmax layer), DBN3 (neural network containing zero DA layer, three RBM layers, and one Softmax layer), SDA4 (neural network containing four DA layers, zero RBM layer, and one Softmax layer), and DBN4 (neural network containing zero DA layer, four RBM layers, and one Softmax layer). Figure 7 shows the training process of the network with 2–4 layers, and the influence of the iteration number on the model error rate is analyzed.

Figure 6 indicates that the recognition accuracy of SDA and DBM models increases with the neural network layer number, and the recognition effect of the SDA model is better than that of the DBM model with the same layers. The recognition accuracy of HNN is higher than that of the single model, which is 97.2%, and with the increase of the number of layers, the training, fine-tuning, and total time of the model are gradually increased. Compared with the SDA4 model, the total time of HNN is reduced by 3.89 min, and the accuracy of HNN is increased by 1.5%. Compared with the DBM4 model, the total time of HNN is extended by 11.52 min, and the accuracy is increased by 1%. Therefore, with the same number of layers, HNN has higher recognition accuracy than the SDA and DBM models, indicating that HNN has inherited strong feature learning from the DA structure and strong feature expression from the RBM structure. Figure 7 suggests that under constant network parameters, the error rate of the training set decreases with the increase of the network layer, decreases with the iteration number, and finally stabilizes. HNN can effectively extract the high-level time-frequency features of the auditory spectrogram, obtaining local optimal parameters through iteration.

**Figure 8:**
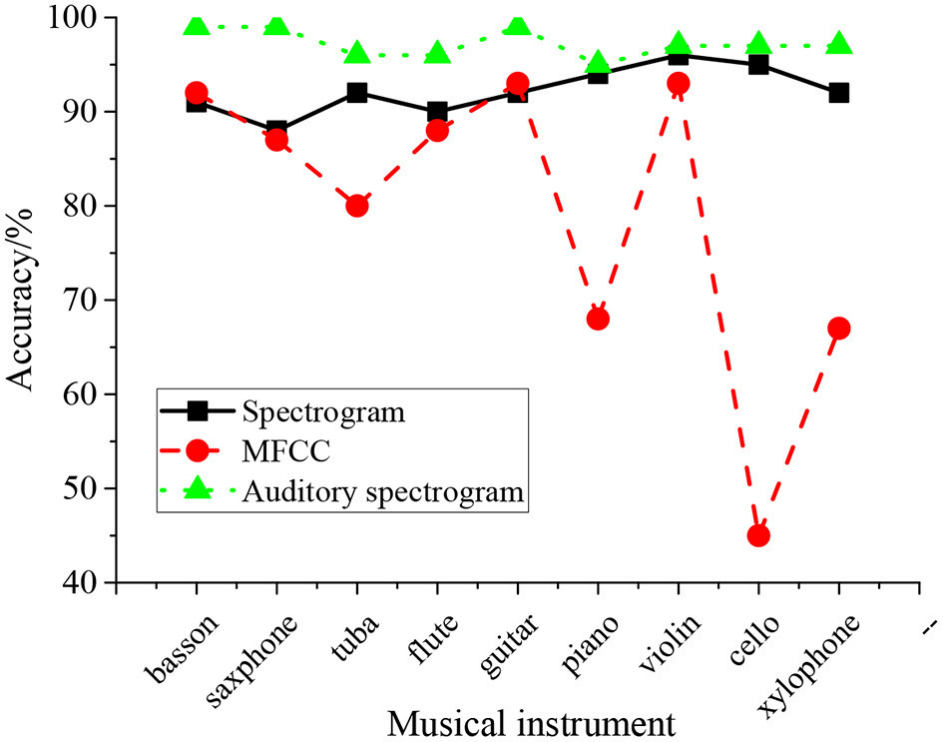
Comparison of different input features.

The spectrogram, MFCC, and auditory spectrogram are input into HNN to verify the influence of input features on the identification accuracy. The results are shown in Figure 8.

Figure 8 illustrates that when the spectrogram is input, the musical identification accuracy of HNN is more than 88%, and the result is stable. When MFCC is input, the identification accuracy fluctuates greatly, and the highest identification accuracy is 93%, while the lowest is 45%. The MFCC extracts information from the source resonant cavity, so the identification accuracy of percussion instruments with the same resonant cavity is poor. When the auditory spectrogram is input into the proposed model, the identification accuracy is about 97% and is higher than the other two input features. Thus, the auditory spectrogram is chosen as the input of HNN for higher identification accuracy for musical instruments.

### QS Results

**Figure 9:**
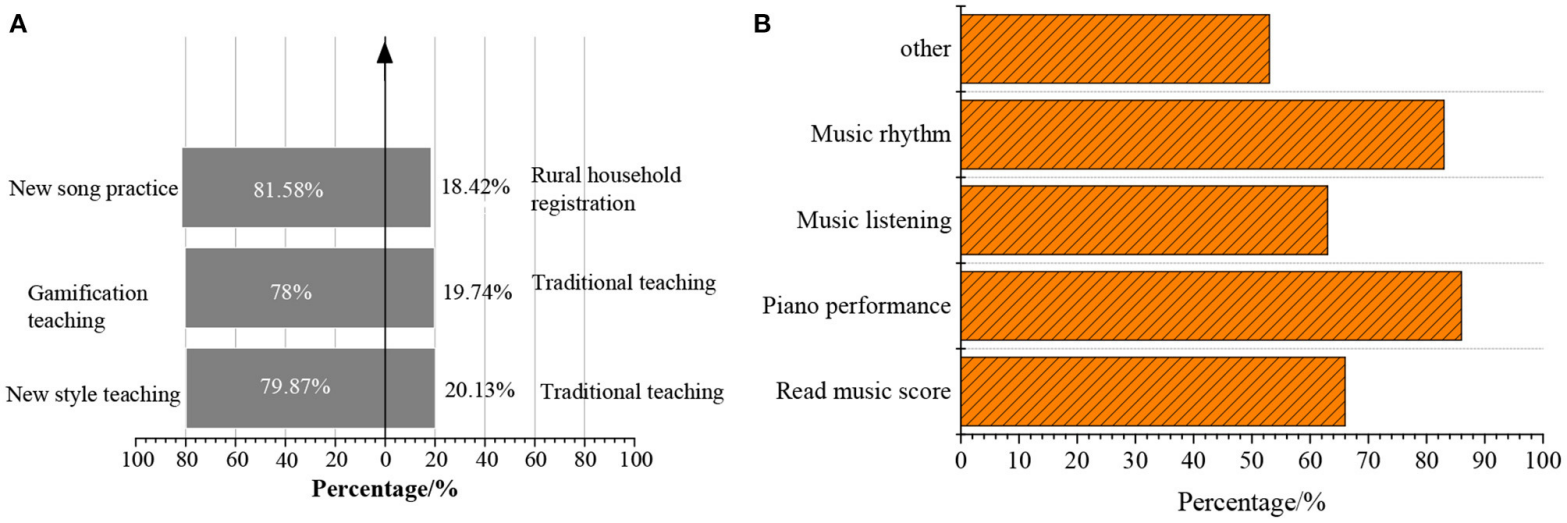
QS result of students. **(A)** QS results statistics. **(B)** Favorite piano music activities of students.

Subsequently, the QS indexes are integrated for different research subjects based on the statistics of the QS results of pre-schoolers (aged between 4 and 6), parents, and teachers. The results of students are shown in Figure 9.

Figure 9 illustrates that 79.87% of the students like innovative teaching, and 80.26% of the students like game-based teaching. The piano education activities that students like, include music rhythm, reading music scores, and music listening. After participating in the new piano education class, 81.58% of the students can take the initiative to practice the piano. Meanwhile, the students have a high learning interest under the new piano education pattern. Thus, the application of the instrument recognition technology based on DL to piano education can improve the learning initiative of students and greatly stimulate the learning interest of the students through situational teaching methods. Additionally, the design of music courses should combine technical characteristics and set up scientific and reasonable course arrangements to better help students learn music courses.

**Figure 10:**
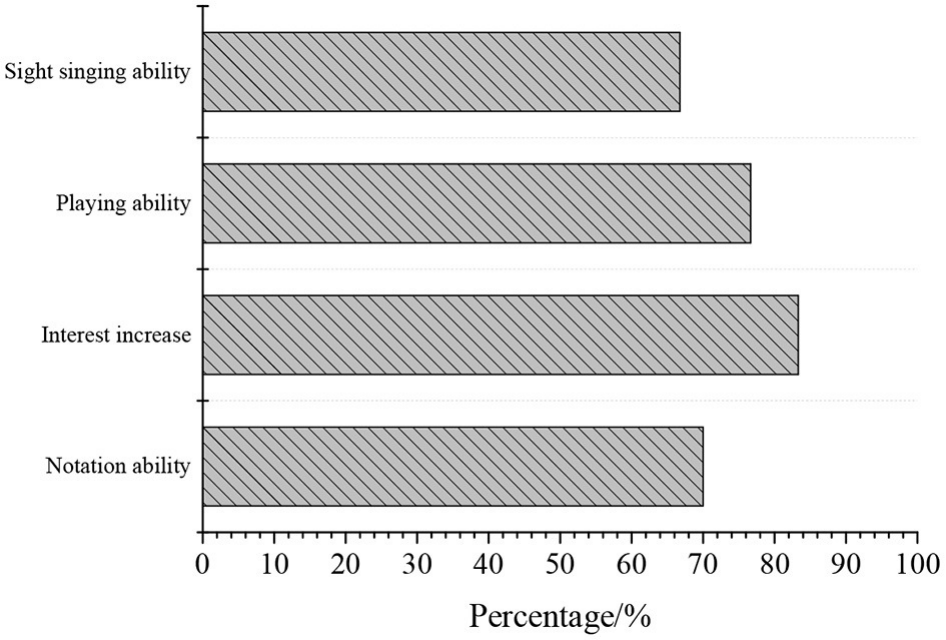
QS result of parents.

**Figure 11:**
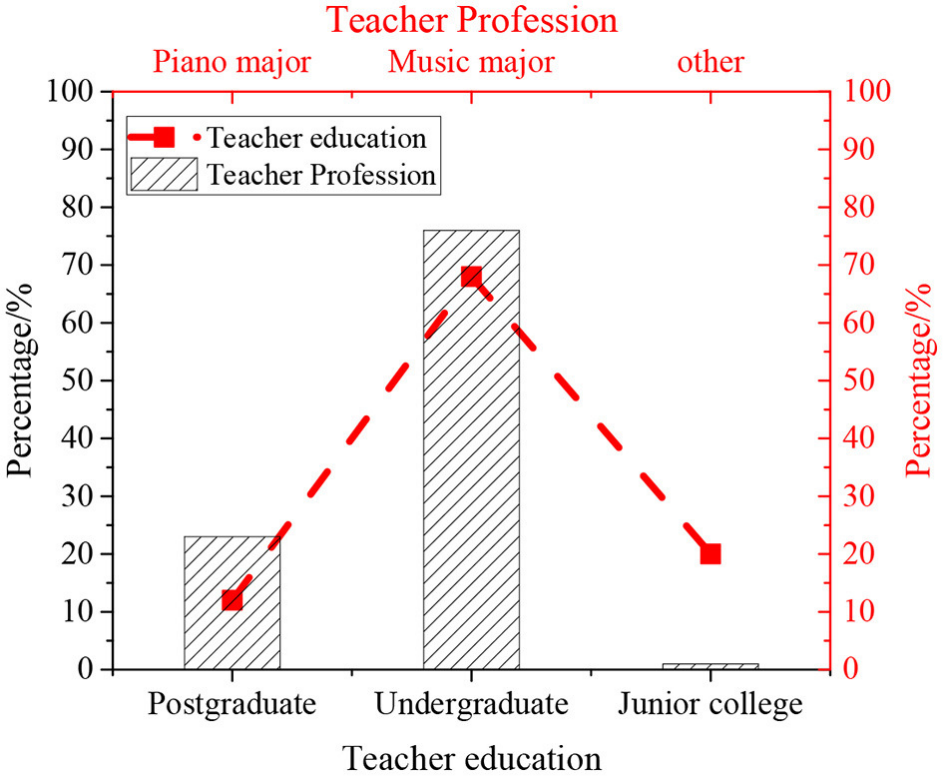
QS result of teachers.

Figure 10 shows the statistical structure of parents, and Figure 11 shows the statistical results of teachers.

The survey results of parents in Figure 10 reveal that 83.33% of the parents believe that after receiving the new piano education class, the interest of the students in learning piano has increased, and their notation ability and solfeggio are improved. The statistical analysis results of the survey of teachers in Figure 11 show that 80% of the piano music teachers have a bachelor's degree or above, and 76% of them are professional music teachers. Therefore, these teachers for piano education have basically received professional systematic teaching and are competent to facilitate piano educational activities for children.

## Discussion

To sum up, the analysis of the proposed instrument recognition technology based on DL showed that the adjustment of neural network structure will affect the recognition accuracy of the model. Under the same number of neural network layers, the proposed model has better recognition accuracy than SDA and DBM models with the same structure. And the increase in the number of layers of the neural network will also reduce the error rate of the training set. The recognition accuracy of different types of music models is different, but the overall recognition accuracy of the instrument recognition model is above 88%. In Pirmoradi et al. (2020), a method for automatically determining the hidden layer and the number of neurons in the DBN was studied. However, the experimental results showed that the input value of the restricted Boltzmann machine could only receive the approximate value to prevent over-fitting, and there was a certain deviation in the results. Ning et al. (2020) introduced the CD-k (k-step contrastive divergence) algorithm to optimize the traditional DBN network, thereby effectively calculating the logarithmic gradient of the Boltzmann machine. Here, experimental results show that the classification accuracy of the proposed method is higher than that of several existing technologies. Specifically, under the comparative analysis of the structures of neural networks with different layers, the optimal network structure is determined so the proposed model can meet the practical application requirements. Meanwhile, the application of the new teaching model to the classroom shows that students, parents, and teachers have a good evaluation of the teaching model, and most students have a high interest in piano learning. Therefore, the proposed new piano education model for children can improve their interest in learning and is welcomed by parents and teachers.

## Conclusion

The purpose of the study was to improve the initiative of learning piano in children. Here, the instrument recognition technology is applied to the piano education of children, and the instrument recognition model is designed with DL technology. Based on the relevant theories of educational psychology, the current content of the piano education of children is designed and innovated to improve their initiative for piano learning and teaching effect. Meanwhile, the musical instrument recognition technology is designed by model combination to improve the feature identification and acquisition effect of the proposed model. The experimental results show that the proposed model can identify the features of music well. The introduction of musical instrument recognition technology in the piano education of children can improve their interest in learning and strengthen the effect of piano education. Specifically, the accuracy of the model for musical instrument recognition is improved by designing the musical instrument recognition model based on the neural network and optimizing the structure of the model. The experimental results show that the instrument recognition model based on DL technology can meet the needs of the piano education and improve the interest of children in piano learning.

Still, there are some shortcomings. The design of the musical instrument recognition model still needs to be further strengthened for higher recognition accuracy. The designed QS is relatively simple, and the scope is insufficient. Besides, the collected QS sample range is concentrated, and under the uneven distribution of educational resources, the ability of students to accept the teaching model may show regional differences. Thus, the results of the QS may be biased. Therefore, in the follow-up study, the instrument recognition model and QS design will be further optimized and updated, and the scope of experimental samples will be expanded to enhance the representativeness of the research results.

## Data Availability Statement

The raw data supporting the conclusions of this article will be made available by the authors, without undue reservation.

## Ethics Statement

The studies involving human participants were reviewed and approved by Communication University of China Ethics Committee. The patients/participants provided their written informed consent to participate in this study. Written informed consent was obtained from the individual(s) for the publication of any potentially identifiable images or data included in this article.

## Author Contributions

The author confirms being the sole contributor of this work and has approved it for publication.

## Conflict of Interest

The author declares that the research was conducted in the absence of any commercial or financial relationships that could be construed as a potential conflict of interest.

## Publisher's Note

All claims expressed in this article are solely those of the authors and do not necessarily represent those of their affiliated organizations, or those of the publisher, the editors and the reviewers. Any product that may be evaluated in this article, or claim that may be made by its manufacturer, is not guaranteed or endorsed by the publisher.

## Appendix

**TABLE A1:** QS (Questionnaire Survey) table.

**QS of intelligent piano based on deep learning**					
Hello! First, thank you very much for your valuable time to participate in this interview. This QS aims to investigate the application of intelligent piano in children's piano education. Filling out this QS may take you about 5 min. There are no correct or wrong answers to each question. Please choose according to your actual situation. We solemnly promise that in this QS, all the information you fill in will only be used for academic research. You don't need to fill in your name. We will keep all the contents in this QS confidential. Thanks again for your cooperation.					
**One**	**Parents and students section**				
1	How old are the students studying the piano?	A. 4 years old	B. 5 years old	C. 6 years old	
2	What kind of textbooks do students like?	A. Intelligent music book		B. Paper music book	
3	What is the student's favorite piano education method?	A. Game-based teaching mode		B. Traditional teaching mode	
4	Do you think students will take the initiative to learn piano?	A. Yes, they will.		B. No, they won't.	
5	What music activities do you think students prefer in intelligent piano class?	A. Read music book	B. Piano performance	C. Music listening and appreciation	D. Music rhythmic activities
6	What do you think is the most important function of intelligent piano for students to learn piano?	A. Accompanied piano practice	B. Remote education	C. Intelligent music book	D. Entertainment
**Two**	**Teachers section**				
7	What's your education level?	A. Bachelor	B. Master	C. Normal universities	
8	What's your major?				
9	How do you use the intelligent piano to arrange lessons in the course?				
10	What do you think are the teaching advantages of intelligent pianos?				
11	How did you solve the problems you encountered in children's intelligent piano education?				
12	Do you have any new teaching concepts in intelligent piano education?				
